# Central hepatectomy versus major hepatectomy for patients with centrally located hepatocellular carcinoma: a systematic review and meta-analysis

**DOI:** 10.1186/s12893-022-01891-7

**Published:** 2023-01-05

**Authors:** Edward Atef Gadallah, Beshoy Effat Elkomos, Ahmed Khalil, Fawzy Salah fawzy, Amr Abdelaal

**Affiliations:** General Surgery Department, Ain Shams University Hospital, Cairo, Egypt

**Keywords:** Centrak hepatectomy, Major hepatectomy, Hepatocellular carcinoma, Centrally located HCC

## Abstract

**Background and aim:**

For those with a centrally located HCC, the two types of liver sectionectomy that can be performed are extended hepatectomy (EH) and central hepatectomy (CH). This meta-analysis aimed to compare the short- and long-term outcomes between patients treated with CH and patients treated with EH for those with centrally located HCC.

**Method:**

We searched PubMed, Scopus, Web of Science, and Cochrane library for eligible studies from inception to 1 April 2022 and a systematic review and meta-analysis were done to compare the outcomes between the two groups.

**Results:**

we included 9 studies with a total of 1674 patients in this study. The pooled results in this meta-analysis showed equal long-term overall survival, Disease-free survival, recurrence and mortality between the two groups (5-year OS, RR = 1.14, 95% CI = 0.96–1.35, P = 0.12; I^2^ = 56%), (5-year DFS, RR = 0.81, 95% CI = 0.61–1.08, P = 0.15; I^2^ = 60%), (Recurrence, RR = 1.04, 95% CI = 0.94–1.15, P = 0.45; I^2^ = 27%), and (Mortality, RR = 0.55, 95% CI = 0.26–1.15, P = 0.11; I^2^ = 0%). In addition to that, no significant difference could be detected in the overall incidence of complications between the two groups (Complications, RR = 0.94, 95% CI = 0.76–1.16, P = 0.57; I^2^ = 0%). However, CH is associated with a remarkable increase in the rate of biliary fistula (Biliary fistula, RR = 1.90, 95% CI = 1.07–3.40, P = 0.03; I^2^ = 0%). And Liver cell failure was higher in the case of EH (LCF, RR = 0.47, 95% CI = 0.30–0.76, P = 0.002; I^2^ = 0%). Regarding the operative details, CH is associated with longer operative time (Time of the operation, Mean difference = 0.82, 95% CI = 0.36, 1.27, P = 0.0004; I^2^ = 57%).

**Conclusion:**

No significant difference in the short and long-term survival and recurrence between CH and MH for CL-HCC. However, CH is associated with greater future remnant liver volume that decreases the incidence of LCF and provides more opportunities for a repeat hepatectomy after tumour recurrence.

**Supplementary Information:**

The online version contains supplementary material available at 10.1186/s12893-022-01891-7.

## Introduction

Hepatocellular carcinoma (HCC) is the fifth-most common cancer globally and the third-highest cause of cancer-related death exceeded only by cancers of the lung and stomach [1]. It is estimated that 782,000 new cases are diagnosed with HCC annually and 600,000 die of this tumour globally each year [2]. treatment modalities are available for patients with local disease including ablation, liver resection, and liver transplantation (LT). However, for those with respectable tumors and tumours underlying liver disease, liver resection offers the best treatment. [3]

Based on Couinaud’s segmental anatomy of the liver, centrally located HCC is defined as tumours located in the middle part of the liver (segments IV, V, or VIII ± I) [4]. For those with a centrally located HCC, the two types of liver sectionectomy that can be performed are, firstly: a major hepatectomy (MH) or an extended hepatectomy (EH) which includes a right/left hemihepatectomy or right/left trisectionectomy and secondly: a central hepatectomy (CH) which involves a left medial sectionectomy, right anterior sectionectomy, or central bisectionectomy (mesohepatectomy).

On one hand, Traditionally, Hemi- or extended hepatectomy is suggested for the treatment of CL-HCC [5]. However, This modality includes the excision of 60–85% of liver parenchyma [6, 7]. Which in turn increases the risk of postoperative liver failure and is associated with higher mortality and morbidity rates [8, 9]. On the other hand, central hepatectomy allows up to 35% parenchymal sparing compared to EH [10]. However, CH has been associated with biliary fistula [11], significant blood loss [6, 12], a longer operative time [6, 13]. This could be explained by the presence of technical challenges related to the presence of two significant parenchymal transection planes in proximity to the hilar bifurcation.

This meta-analysis aimed to compare the short- and long-term outcomes including overall survival, recurrence rate and complications between patients treated with CH and patients treated with Hemi-/extended hepatectomy for those with centrally located HCC.

## Patients and methods

### Search strategy

We searched the database including (PubMed, Scopus, the Cochrane Library and Web of Science) from inception to 1 April 2022 using the following search terms: major hepatectomy and Mesohepatectomy or central Hepatectomy and hepatocellular carcinoma. In addition to that Google Scholar was searched to detect the presence of any missing articles. All the studies that met our inclusion criteria were included and the manuscripts were fully reviewed. All the included studies were reviewed by two authors independently (Gadallah, E. A. & Elkomos, B. E.).

### Inclusion and exclusion criteria

The eligible studies included the following: (1) randomized controlled trials and prospective or retrospective cohort studies; (2) the target population were patients with hepatocellular carcinoma; (3) studies designed to compare central hepatectomy versus extended hepatectomy for hepatocellular carcinoma; (4) studies providing a sufficient description of the methods and baseline characteristics, and (5) the main outcomes were patient overall survival, disease-free survival for both central and major hepatectomy. The following types of studies were not included in our study: (1) unrelated or in vitro studies; (2) reviews, case reports and case series; (3) patients diagnosed with liver cancers other than hepatocellular carcinoma: (3) studies missing a comparison group.

### Outcomes of interest

We assessed overall survival for central and extended hepatectomy for hepatocellular carcinoma as a primary outcome (1, 2, 3, 4, 5-year OS). in addition to that, we assessed 5 secondary outcomes including disease-free survival (1, 2, 3, 4, 5-year DFS), recurrence, early postoperative mortality, complications (liver cell failure, biliary fistula, wound infection and ascites), operative details (the time of the operation, the blood loss during the operation, blood transfusion and hospital stay after operation).

### Data extraction

We extracted data on study characteristics (author, year of publication, country of operation, type of study and sample size), patient characteristics (age, sex, child score, virology and cirrhosis), tumour biology (tumour size, tumour number and vascular invasion), operative details (the time of the operation, the blood loss during the operation, blood transfusion, hospital stay after operation and resection margin), Patients outcome (overall survival, disease-free survival, recurrence and mortality) and complications (overall incidence of complications, liver cell failure, biliary fistula and wound infection). The data were extracted by 2 investigators (Gadallah, E. A. & Elkomos, B. E.) independently.

### Statistical analysis

Cochrane Handbook of Systematic Reviews of Interventions [14] which is recommended by the Cochrane Collaboration was used as a guide while conducting this meta-analysis. For all the results included, the pooled risk ratios (RRs) and their corresponding 95% confidence intervals (CIs) were calculated with fixed effects models. However, if there was moderate or considerable heterogeneity (I^2^ > 40), random effects models were used to solve the heterogeneity between studies. Review Manager 5.4 (Cochrane Collaboration, Oxford, United Kingdom) was used for all calculations in this meta-analysis.

### Assessment of publication bias and heterogeneity

Funnel plots were generated so that we could visually inspect for publication bias. Statistical heterogeneity was assessed with forest plots and the inconsistency statistic (I^2^). An I^2^ value of 40% or less corresponded to low heterogeneity. Statistical significance was considered at P < 0.05.

## Results

### Characteristics and quality assessment of eligible studies

**Fig. 1:**
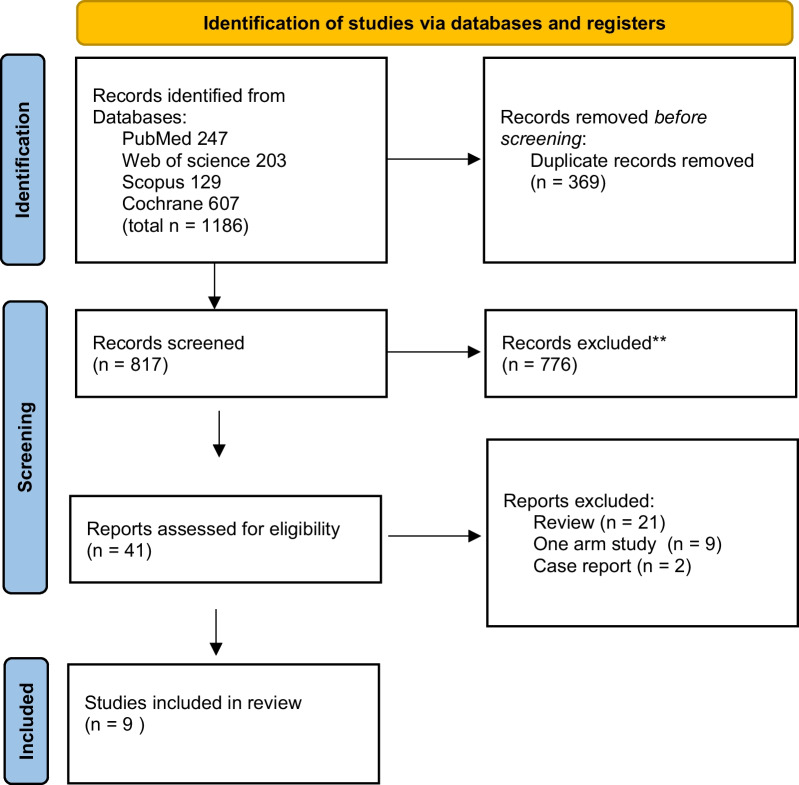
PRISMA flow diagram

As shown in the flow diagram (Fig. 1),1186 articles were revealed using a combination of the following words: major hepatectomy and Mesohepatectomy or central Hepatectomy and hepatocellular carcinoma. After careful selection based on our eligibility criteria, 9 studies with 1674 patients were included in the meta-analysis. All the included studies were cohort studies. The studies were conducted in four different countries (China, Taiwan, Japan and Mongolia).

Patients’ characteristics (age, sex, child score, virology and cirrhosis), and tumour biology (tumour size, tumour number and vascular invasion) were comparable between the two groups in all studies (Table 1).

**Table 1:** Basic data of the included studies

Author and Publication year	Country	Study design	Study period	Arm	Sample size (n)	Age (yr)	Gender: M/F(n)	Child score(A/B/C)	Virology (HBV/HCV/Both)	Cirrhosis (n/%)	ICG-R15 (%)	Tumor size (cm)	Tumor number (S/M)	Vascular invasion (%)	Resection margin (< 1 cm/ > 1 cm)
Wu, 1999 [15]	Taiwan	Retrospective Cohort	1987–1997	CH	15	53.0 ± 11.6 ^a^	15/0	14/1/0	13/2/0	11–	N/A	12.8	N/A	N/A	N/A
				EH	25	N/A	N/A	N/A	N/A	N/A	N/A	N/A	N/A	N/A	N/A
Hu, 2003 [12]	Taiwan	Cohort	1993–1999	CH	52	N/A	N/A	N/A	24(HCV)	24	17.4 ± 1.5^a^	< 9	47/5	12	22/30
				EH	63	N/A	N/A	N/A	13(HCV)	24	12.4 ± 2.2^a^	< 9	50/13	26	16/47
Cheng, 2012 [16]	Taiwan	Cohort	1999–2005	CH	63	58(50–66)^b^	50/13	56/7/0	43/13/0	30 (47.6%)	8.57 (5.20–13.53)^b^	6.50 (5.50–8.50)^b^	N/A	30 (47.6%)	50//4
				EH	41	61(50–68.5)^b^	32/9	37/3/0	27/8/0	15 (36.6%)	8.14 (5.88–14.48)^b^	8.00 (5.50–10.25)^b^	N/A	19 (46.3%)	30//8
Chen, 2014 [17]	China	Cohort	2002–2008	CH	118	56.4 ± 12.3^a^	96/22	6.7 ± 1.2	100/15/0	106	5.3 ± 2.0	8.6 ± 2.4	N/A	30	39/79
				EH	80	N/A	67/13	N/A	69/10/0	68	N/A	N/A	N/A	24	13/67
Yang, 2014 [18]	China	Retrospective Cohort	2002–2012	CH	350	N/A	298/52	298/52/0	315 (HBV)	281	N/A	N/A	161/189	195	144/206
				EH	346	47.5 ± 11.8^a^	289/57	284/62/0	303 (HBV)	272	N/A	8.1 ± 5.3^a^	141/205	194	133/213
Chinburen, 2015 [19]	Mongolia	Retrospective Cohort	2003–2012	CH	45	59.8 ± 8.5^a^	23/22	30/0/0	14/21/0	N/A	N/A	5.9 ± 2.2^a^	37/8	N/A	N/A
				EH	24	55.4 ± 9.2 ^a^	12//12	14/1/0	8/7//0	N/A	N/A	7.2 ± 2.1 ^a^	21/3	N/A	N/A
Chen, 2017 [20]	Taiwan	Retrospective Cohort	2007–2010	CH	15	62 ± 14^a^	12//3	N/A	10/3//1	3	8.94 ± 5.08^a^	4.71 ± 2.25^a^	N/A	5	N/A
				MH	33	60 ± 13^a^	22/11	N/A	18//5//2	15	8.34 ± 4.31^a^	5.28 ± 4.17^a^	N/A	15	N/A
Li, 2018 [13]	China	Retrospective Cohort	200–2016	CH	87	53.0 ± 7.8^a^	63/24	N/A	74 (HBV)	80	7.3 ± 1.9^a^	4.2 ± 0.9^a^	N/A	10 (11.5%)	N/A
				MH	84	54.4 ± 9.2^a^	66/18	N/A	70 (HBV)	67	7.3 ± 2.3^a^	4.1 ± 1.0 ^a^	N/A	10 (11.9%)	N/A
Orimo, 2021 [21]	Japan	Retrospective Cohort	200–2019	CH	132	68 (39–86)^b^	112/20	N/A	40/39/0	N/A	11.6 (2.9–86.7)^b^	4.5 (1.2–15.2)^b^	91/41	N/A	N/A
				MH	101	65 (33–85) ^b^	85/16	N/A	45/22/0	N/A	10.8 (1.4–54.0)^b^	6.5 (0.6–22.5) ^b^	65/36	N/A	N/A

^a^The results are presented as means and standard deviation

^b^The results are presented as median and range

Table 2 summarizes the outcomes for CH and EH for HCC.

**Table 2:** Outcomes for central and extended hepatectomy for hepatocellular carcinoma

Outcomes	Studies (n)	Patients (n)	Effect estimate [RR/MD (95% CI)]	Heterogeneity	Test for overall effect	Favour group
Overall survival						
1-year	8	1634	1.00 [0.96, 1.04]	I^2^ = 16% (P = 0.31)	Z = 0.10 (P = 0.92)	None
2-year	6	855	1.14 [1.06, 1.23]	I^2^ = 21% (P = 0.27)	Z = 3.46 (P = 0.0005)	CH
3-year	7	1530	1.13 [0.97, 1.33]	I^2^ = 71% (P = 0.002)	Z = 1.56 (P = 0.12)	None
4-year	5	765	1.31 [1.16, 1.48]	I^2^ = 6% (P = 0.36)	Z = 4.38 (P < 0.0001)	CH
5-year	7	1565	1.14 [0.96, 1.35]	I^2^ = 56% (P = 0.03)	Z = 1.54 (P = 0.12)	None
Disease free survival						
1-year	8	1605	1.03 [0.92, 1.15]	I^2^ = 50% (P = 0.05)	Z = 0.49 (P = 0.63)	
2-year	5	765	1.04 [0.90, 1.20]	I^2^ = 29% (P = 0.23)	Z = 0.48 (P = 0.63)	
3-year	7	1447	1.19 [0.91, 1.56]	I^2^ = 74% (P = 0.0007)	Z = 1.26 (P = 0.21)	
4-year	5	765	0.92 [0.75, 1.13]	I^2^ = 37% (P = 0.18)	Z = 0.79 (P = 0.43)	
5-year	7	1565	0.81 [0.61, 1.08]	I^2^ = 60% (P = 0.02)	Z = 1.43 (P = 0.15)	
Recurrence	4	1081	1.04 [0.94, 1.15]	I^2^ = 27% (P = 0.25)	Z = 0.76 (P = 0.45)	
Mortality	8	1626	0.55 [0.26, 1.15]	I^2^ = 0% (P = 0.91)	Z = 1.58 (P = 0.11)	
Complications						
Overall	7	1270	0.94 [0.76, 1.16]	I^2^ = 0% (P = 0.48)	Z = 0.57 (P = 0.57)	
Liver cell failure	6	1415	0.47 [0.30, 0.76]	I^2^ = 0% (P = 0.52)	Z = 3.10 (P = 0.002)	CH
Biliary fistula	7	1455	1.90 [1.07, 3.40]	I^2^ = 0% (P = 0.85)	Z = 2.18 (P = 0.03)	EH
Ascites	4	1084	1.95 [1.00, 3.78]	I^2^ = 0% (P = 0.88)	Z = 1.97 (P = 0.05)	None
Wound infection	5	1282	0.77 [0.39, 1.52]	I^2^ = 0% (P = 0.79)	Z = 0.76 (P = 0.44)	
Operative details						
Time of the operation	4	328	0.82 [0.36, 1.27]	I^2^ = 57% (P = 0.07)	Z = 3.53 (P = 0.0004)	
Blood loss	3	280	40.87 [− 8.81, 90.54]	I^2^ = 13% (P = 0.32)	Z = 1.61 (P = 0.11)	
blood transfusion	3	224	269.54 [− 169.28, 708.35]	I^2^ = 78% (P = 0.01)	Z = 1.20 (P = 0.23)	
Hospital stay	3	280	− 2.17 [− 5.56, 1.22]	I^2^ = 83% (P = 0.003)	Z = 1.25 (P = 0.21)	

### Primary outcome

#### Overall survival

**Fig. 2:**
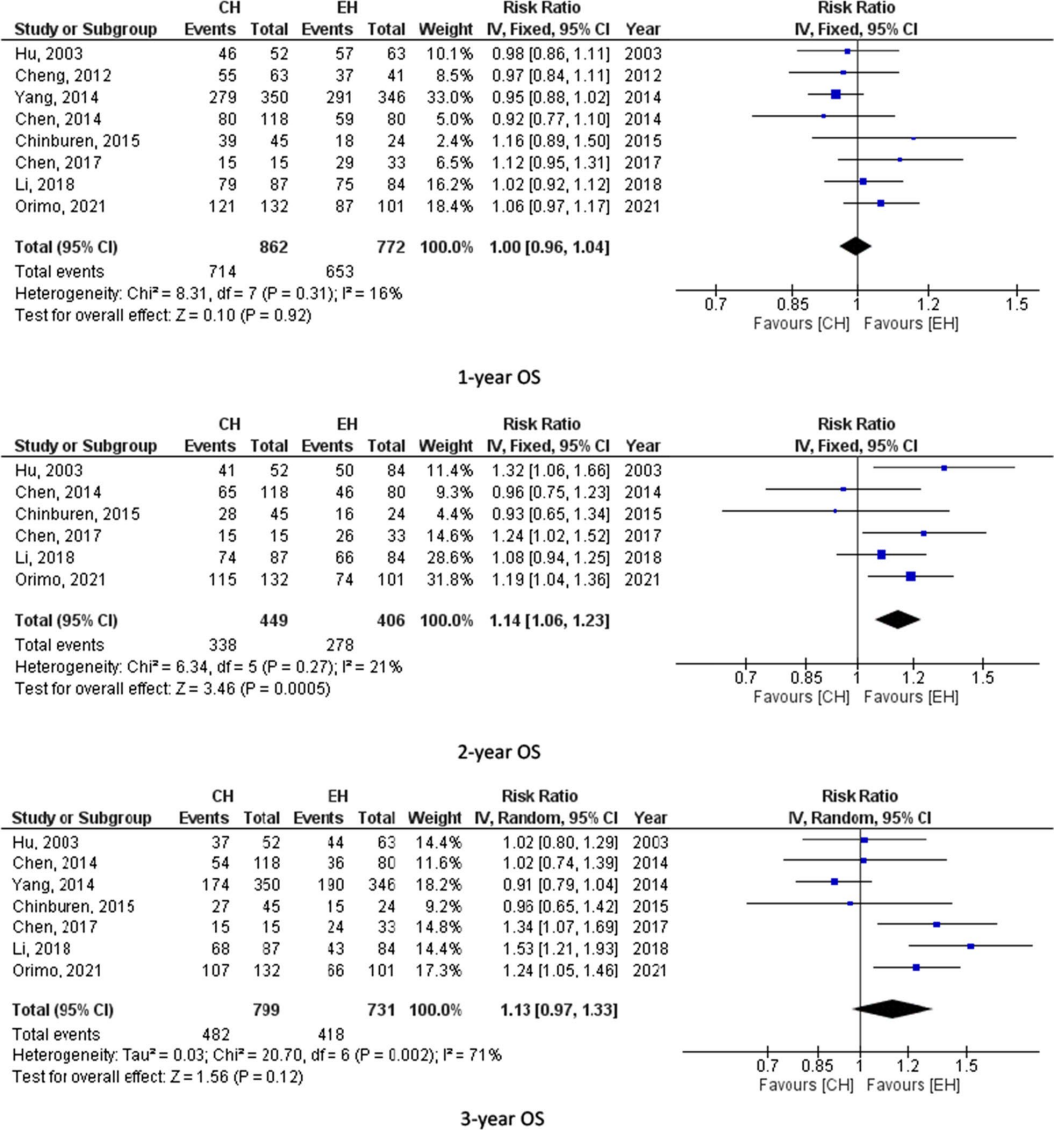

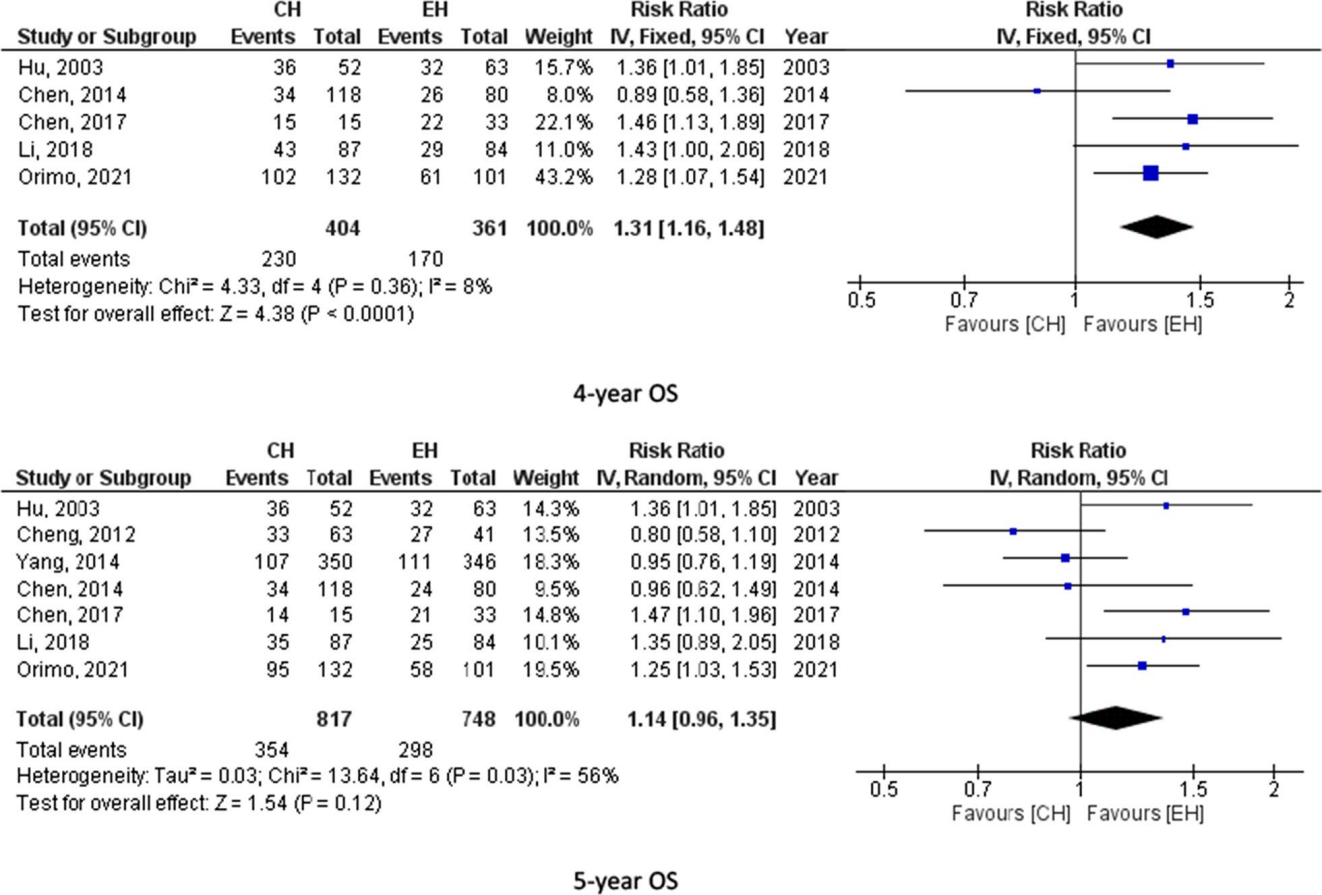
overall survival for CH and EH

Eight studies (1634 participants) assessed 1-year OS, 7 studies (1530) reported 3-year OS and 7 studies (1565) calculated 5 year-OS. The pooled results from these studies showed equal overall survival for those who underwent central hepatectomy and extended hepatectomy as follows (1-year OS, RR = 1.00, 95% CI = 0.96–1.04, P = 0.92; I^2^ = 16%), (3-year OS, RR = 1.13, 95% CI = 0.97–1.33, P = 0.12; I^2^ = 71%) and (5-year OS, RR = 1.14, 95% CI = 0.96–1.35, P = 0.12; I^2^ = 56%). 5-year OS was 43.3% for CH and 39.8% for EH. However, the pooled results for the 2 and 4-year overall survival showed possible improvement in the overall survival for those who underwent extended hepatectomy (2-year OS, RR = 1.14, 95% CI = 1.06–1.23, P = 0.0005; I^2^ = 21%) and (4-year OS, RR = 1.31, 95% CI = 1.16–1.48, P < 0.0001; I^2^ = 8%) as shown in 6 studies (855) for 2-year OS and 5 studies (765) for the 4-year OS. 4-year OS was 56.9% for CH and 47% for EH. Figure 2 summarizes 1-, 2-, 3-, 4-and 5-year OS for CH and EH recipients.

### Secondary outcomes

#### Disease free survival

**Fig. 3:**
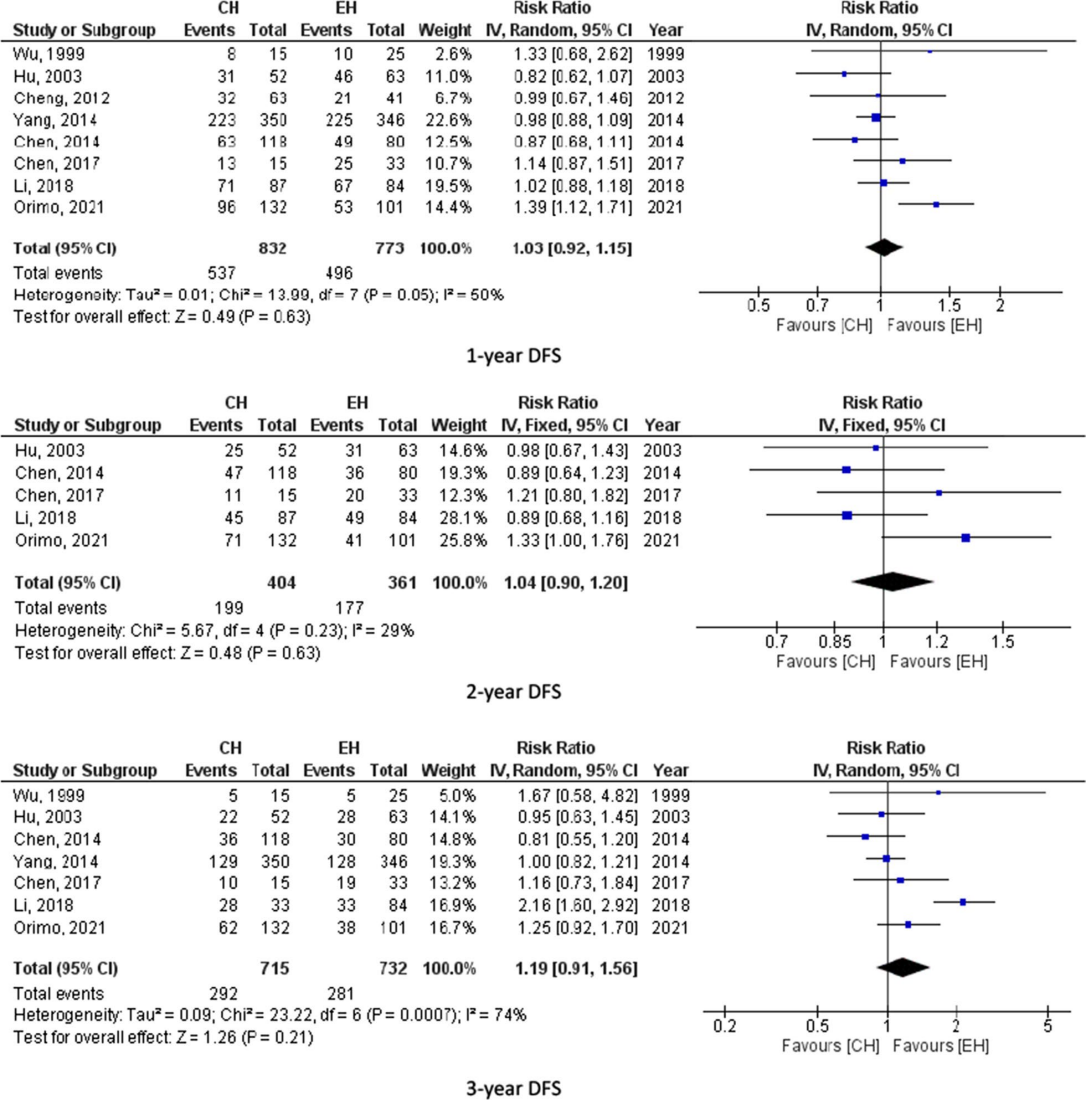

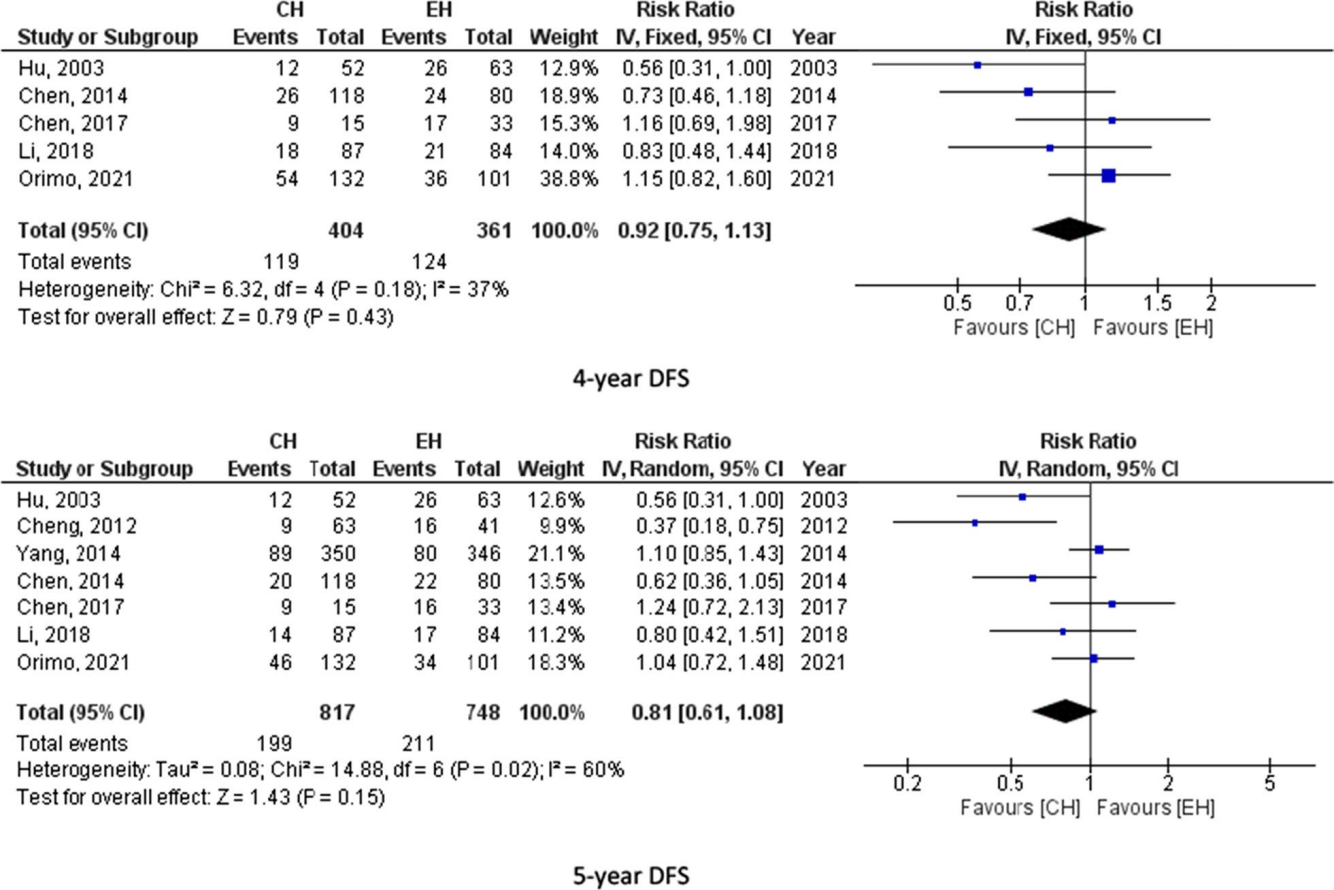
Disease free survival for CH and EH

Eight studies (1605 participants) reported 1-year DFS, 5 studies (765 participants) assessed 2-year DFS, 7 studies (1447 participants) reported 3-year DFS, 5 studies (765 participants) calculated 4-year DFS and 7 studies (1565 participants) assessed 5-year DFS. The pooled results from these studies showed no significant difference between CH and EH. For instance, 5-year DFS was 24.4% for CH and 28.2% for EH. Figure 3 summarizes 1-, 2-, 3-, 4- and 5-year DFS for CH and EH recipients.

#### Recurrence

**Fig. 4:**
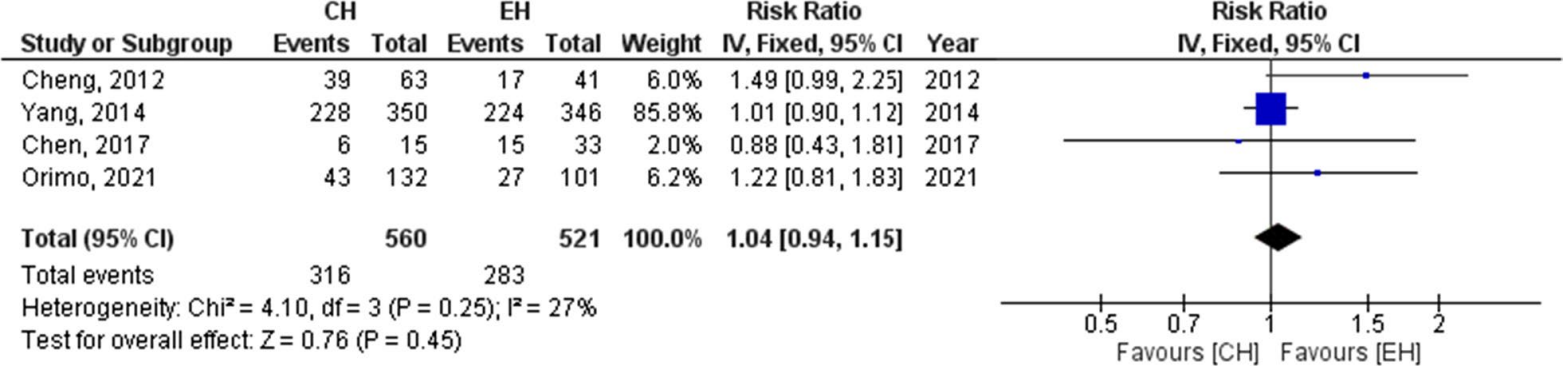
Recurrence for CH and EH

Regarding the recurrence, after hepatectomy as reported by 4 studies (1081 patients), no significant difference could be detected between the groups (Recurrence, RR = 1.04, 95% CI = 0.94–1.15, P = 0.45; I^2^ = 27%) Fig. 4. According to the pooled results of these studies, the recurrence was 56.4% for those who underwent CH and 54.3% for those who underwent EH.

#### Mortality

In addition to that, as reported by 8 studies (1626), the early post-operative mortality during the first three months after surgery was nearly equal for the two modalities. It was an average of 2% for both groups. (Mortality, RR = 0.55, 95% CI = 0.26–1.15, P = 0.11; I^2^ = 0%) Additional file 1: Fig. S1.

#### Complications

Turning to post-operative complications, no remarkable difference in the total incidence of postoperative complications in the two groups. As reported by 7 studies (1270 patients), the pooled results showed a 19.9% complication rate for CH and 19.8% for EH. (Complications, RR = 0.94, 95% CI = 0.76–1.16, P = 0.57; I^2^ = 0%) Additional file 2: Fig. S2.

#### Liver cell failure

On one hand, liver cell failure was calculated for the two groups in 6 studies (1415 participants) and its incidence was higher in the EH group 5.3% in comparison to 3% in the CH group. (LCF, RR = 0.47, 95% CI = 0.30–0.76, P = 0.002; I^2^ = 0%) Additional file 3: Fig. S3.

#### Biliary fistula

On the other hand, the biliary fistula was reported in 7 studies (1455 patients) and the rate was higher in CH group 5% in comparison to 2.5% for EH group. (Biliary fistula, RR = 1.90, 95% CI = 1.07–3.40, P = 0.03; I^2^ = 0%) Additional file 4: Fig. S4.

#### Ascites

According to 4 studies (1084 participants), no significant difference could be detected in the rate of post-operative ascites in the two groups. (Ascites, RR = 1.95, 95% CI = 1.00–3.78, P = 0.05; I^2^ = 0%) Additional file 5: Fig. S5.

#### Wound infection

Moreover, as reported by 5 studies (1282 patients), no remarkable difference in the incidence of wound infection for the two modalities. (Wound infection, RR = 0.77, 95% CI = 0.39–1.52, P = 0.44; I^2^ = 0%) Additional file 6: Fig. S6.

#### Operative details

##### Time of operation

Turning to the duration of the operation, according to 4 studies (328 patients), the average time for surgery was longer in CH than in EH. (Time of the operation, Mean difference = 0.82, 95% CI = 0.36, 1.27, P = 0.0004; I^2^ = 57%). Additional file 7: Fig. S7.

##### Blood loss during operation

According to three studies (280 participants), no remarkable difference could be detected in the blood loss during the operation for both methods (Blood loss, Mean difference = 40.87, 95% CI = − 8.81, 90.54, P = 0.11; I^2^ = 13%). Additional file 8: Fig. S8.

##### Blood transfusion

In addition to that, as reported by three of the included studies (224 participants), the average amount of blood transfusion was similar for the two groups. (Blood transfusion, Mean difference = 269.54, 95% CI = − 169.28, 708.35, P = 0.78; I^2^ = 23%). Additional file 9: Fig. S9.

##### Hospital stay

In addition to that, according to the pooled results from three studies (280 participants), the two groups had the equal time of hospitalization. (Hospital stay, Mean difference = − 2.17, 95% CI = − 5.56, 1.22, P = 0.21; I^2^ = 83%). Additional file 10: Fig. S10.

#### Publication bias assessment

The funnel plot analysis demonstrated a symmetrical appearance. However, it was not reliable because only nine studies were included.

## Discussion

Hepatocellular carcinoma (HCC) is the primary liver cancer derived from hepatocytes and accounts for 85–90% of all primary liver cancers [2]. Liver resection, ablation and transplantation are the curable treatment options for this tumour and according to BCLC treatment recommendations for HCC, liver resection offers the best treatment for HCC without underlying liver disease [3]. The two types of liver resection that can be performed for centrally located HCC are major hepatectomy (MH) or extended hepatectomy (EH) and central hepatectomy (CH) which was first performed by McBride and Wallace 1972 as a treatment for gall bladder cancer and intended as en bloc excision of the Couinaud’s segments 4, 5, 8 ± 1.

To begin with overall survival after hepatectomy, according to a recent systematic review, the 5-year OS after hepatectomy for HCC ranged from 30% to 61.4% [22]. On one hand, some studies reported better overall survival for those who underwent central hepatectomy [20, 21]. This has been explained by the increased liver volume preservation which might be associated with favourable OS [20] and as reported by Lee SY, the 5-year OS for those who underwent CH for HCC ranged from 31.7% to 66.8% [7]. However, on the other hand, other studies said that the overall survival was equal between the two modalities [17, 19]. In our meta-analysis a trivial improvement could be detected in the 2- and 4-years OS for those who underwent CH. However, no significant difference could be detected in the 5-year OS between the two modalities with a 5-year OS of 43.3% for CH and 39.8% for EH. In addition to that, DFS was similar in the two groups.

Although the early postoperative mortality rate of liver resection has been reduced to a few per cent in recent case series, its overall morbidity rate is reported to range from 4.1% to 47.7% [23, 24]. The causes for early post-operative mortality are haemorrhage, liver failure leading to ascites and hepatic encephalopathy, pulmonary infection/ pleural effusion/empyema, urinary tract infection, sepsis, upper gastrointestinal bleeding, renal failure, stroke, deep vein thrombosis, wound infection, intra-abdominal abscess and intestinal perforation. In our study, the incidence of this mortality was similar in the two groups.

Regarding the recurrence rate of HCC, as reported by some studies, central hepatectomy increases the chance of a future repeat resection. [25, 26] However, according to the pooled results from the included studies, no remarkable difference could be detected in the recurrence rate between the two types of liver resection. In addition to that as reported by Orimo et al. [21]. hepatectomy that was more in the CH group not because liver recurrence was more common in the CH group, but because the sufficient remnant liver that was preserved in the CH group could be removed after recurrence.

Post-liver resection complications tend to be severe and the risk factors for complications after liver resection depend on the pathological background of the liver itself [27]. These complications include liver cell failure, biliary fistula, ascites, surgical site infection, pneumonia and respiratory distress. Our meta-analysis showed that the overall incidence of complications was comparable between the two modalities.

To begin with liver cell failure, is the most serious complication after liver resection and can be life-threatening [28]. with estimated mortality ranging between 60 and 80% depending on the cause and the experience of the clinical department to which the patient is referred [29, 30]. And as reported by Van Den Broek et al., the incidence of post-resection liver cell failure after partial hepatic resection ranges from 0.7 to 9.1% and the key events in the pathogenesis are inadequate quantity or quality of residual liver mass [31]. According to the pooled results of the included studies, the incidence of postoperative liver cell failure was significantly higher in those who underwent major hepatectomy. This could be attributed to the fact that major hepatectomy is associated with the removal of 60–85% of liver parenchyma. [6, 7]

Regarding bile leakage after liver resection, it is one of the most frequently reported intra-abdominal complications [32]. And according to the American College of Surgeons National Surgical Quality Improvement Program (ACS NSQIP), the incidence of biliary leakage after liver resection has been reported approximately 7% [33, 34]. In our study, a higher incidence of the biliary fistula was detected for those who underwent central hepatectomy. This has been explained by the presence of two transection planes and exposure of the hepatic hilum [35].

Ascites, which means pathological accumulation of fluid within the abdominal cavity and the word “ascites” is derived from the Greek word “asks,” which means a bag or sack [36], is a common complication in patients who exhibit liver dysfunction or cirrhosis after liver resection [37]. This complication has been explained by the increase in portal flow resistance at the sinusoidal level due to a reduction in the volume of the portal vascular bed [38]. And the acute phase after liver resection causes oedema in the interstitial organ space, which leads to increased portal flow resistance [37]. According to the pooled results of included studies, no significant difference could be detected in the rate of post-operative ascites in the two groups.

surgical site infections are common after all types of surgery and are classified into superficial, deep incisional, and organ/space surgical site infections [39]. According to the CDC, SSIs are infections that occur within 30 days of surgery or one year if an implant is present [40]. In our study, no difference in the incidence of SSI could be detected between the two types of liver resection.

Turning to the operative details, many studies reported that central hepatectomy is associated with greater operative blood loss and the need for operative blood transfusion [6, 12] and this was explained by technical complexity, which is the result from the presence of two parenchymal transection planes in proximity to the hilar bifurcation [5, 15]. As a result, it requires challenging handling of the right hepatic vein exposed along the right section plane, middle hepatic vein at its distal end, biliary confluence, and first- and second-order portal pedicles. However, the pooled results showed no remarkable difference between CH and MH in terms of blood loss and blood transfusion during the operation. It is worth mentioning that the operative time was longer in the case of central hepatectomy and as we mentioned earlier, this is because of the technical complexity that is associated with central hepatectomy.

To our knowledge it is the largest meta-analysis to compare the two types of resection for HCC as all the studies that were comparing the outcomes between the two modalities were included. However, we have to admit that all the included studies were cohort studies which are considered a limitation in our study because no randomized controlled trials could be found.

## Conclusion

This study showed no significant difference in the short and long-term survival and recurrence between CH and MH for CL-HCC. However, CH is associated with greater future remnant liver volume that decreases the incidence of LCF and provides more opportunities for a repeat hepatectomy after tumour recurrence.

## Supplementary Information

**Additional file 1: Fig. S1**. Mortality for CH and EH.

**Additional file 2: Fig. S2**. Post-operative complications for CH and EH.

**Additional file 3: Fig. S3**. Liver cell failure for CH and EH.

**Additional file 4: Fig. S4**. Biliary fistula for CH and EH.

**Additional file 5: Fig. S5**. Ascites for CH and EH.

**Additional file 6: Fig. S6**. Wound infection for CH and EH.

**Additional file 7. Fig. S7**. Operative time for CH and EH.

**Additional file 8. Fig. S8**. Blood loss for CH and EH.

**Additional file 9. Fig. S9**. Blood transfusion for CH and EH.

**Additional file 10: Fig. S10**. Hospital stay for CH and EH.

## Data Availability

All data generated or analysed during this study are included in this published article and its Additional files (all the studies that were included in this meta-analysis are included in Additional files).
