# Supplementary figures and images for: Central hepatectomy versus major hepatectomy for patients with centrally located hepatocellular carcinoma: a systematic review and meta-analysis
Source: BMC Surg. 2023 Jan 5;23:2. doi: 10.1186/s12893-022-01891-7 (PMC9814185; doi:10.1186/s12893-022-01891-7)

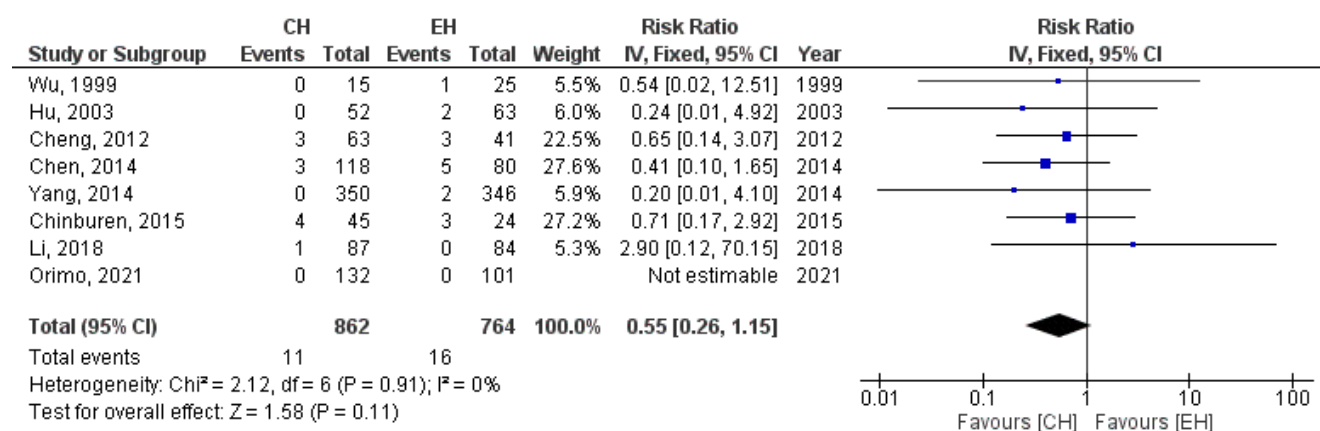

supplementary figure 1 : Mortality for CH and EH

Supplement: Supplementary file 1 — Additional file 1: Fig. S1. Mortality for CH and EH. [file 12893_2022_1891_MOESM1_ESM.pdf]

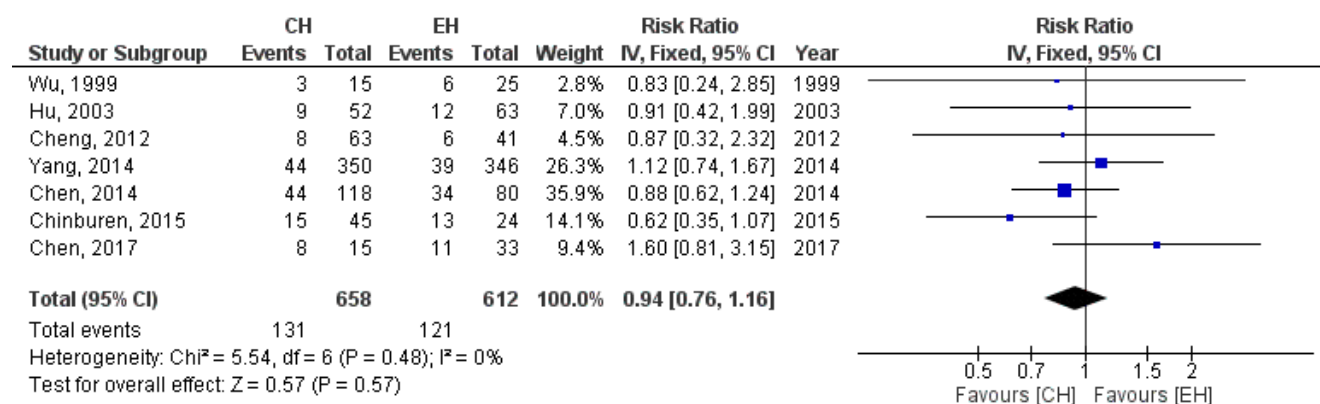

supplementary figure 2: Post-operative complications for CH and EH

Supplement: Supplementary file 2 — Additional file 2: Fig. S2. Post-operative complications for CH and EH. [file 12893_2022_1891_MOESM2_ESM.pdf]

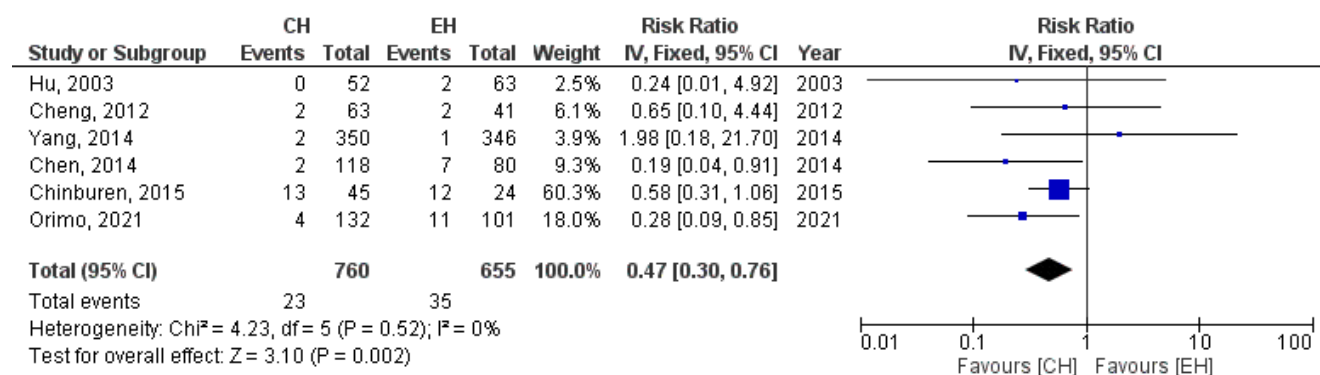

supplementary figure 3: Liver cell failure for CH and EH

Supplement: Supplementary file 3 — Additional file 3: Fig. S3. Liver cell failure for CH and EH. [file 12893_2022_1891_MOESM3_ESM.pdf]

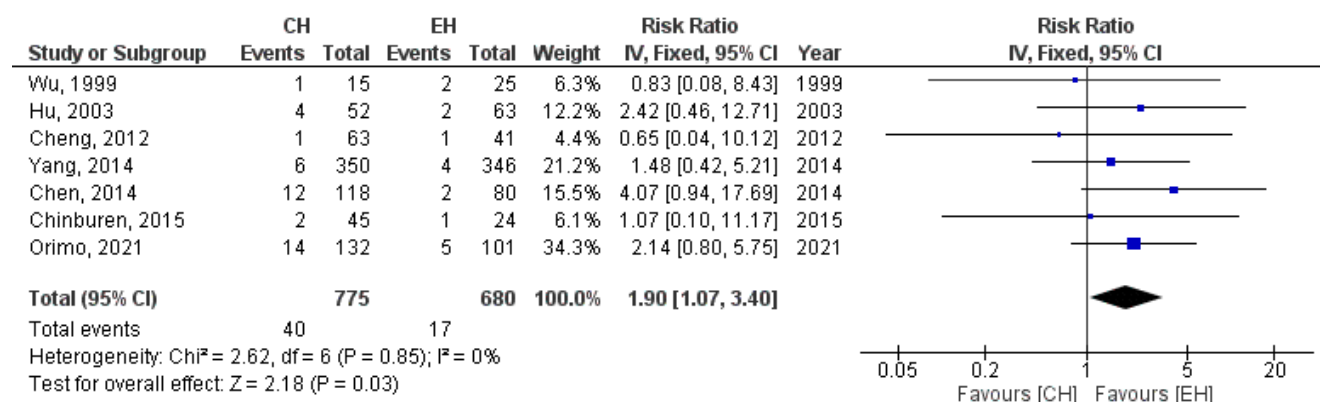

supplementary figure 4: Biliary fistula for CH and EH

Supplement: Supplementary file 4 — Additional file 4: Fig. S4. Biliary fistula for CH and EH. [file 12893_2022_1891_MOESM4_ESM.pdf]

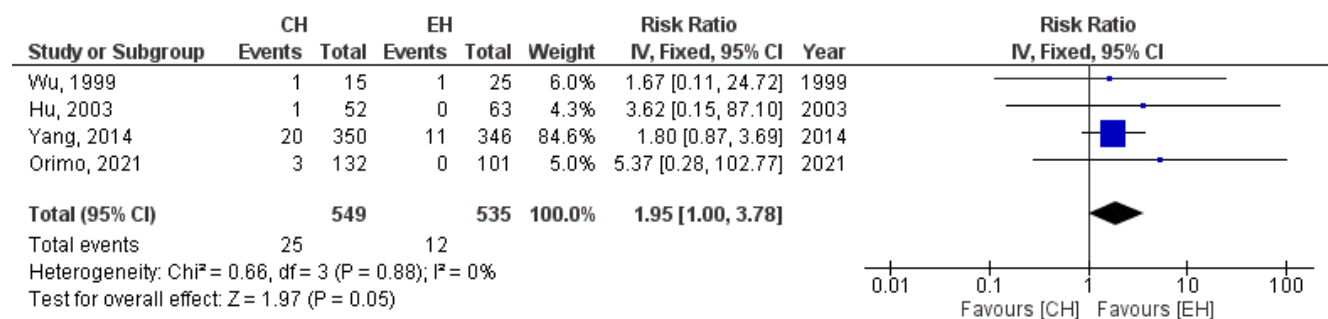

supplementary figure 5: Ascites for CH and EH

Supplement: Supplementary file 5 — Additional file 5: Fig. S5. Ascites for CH and EH. [file 12893_2022_1891_MOESM5_ESM.pdf]

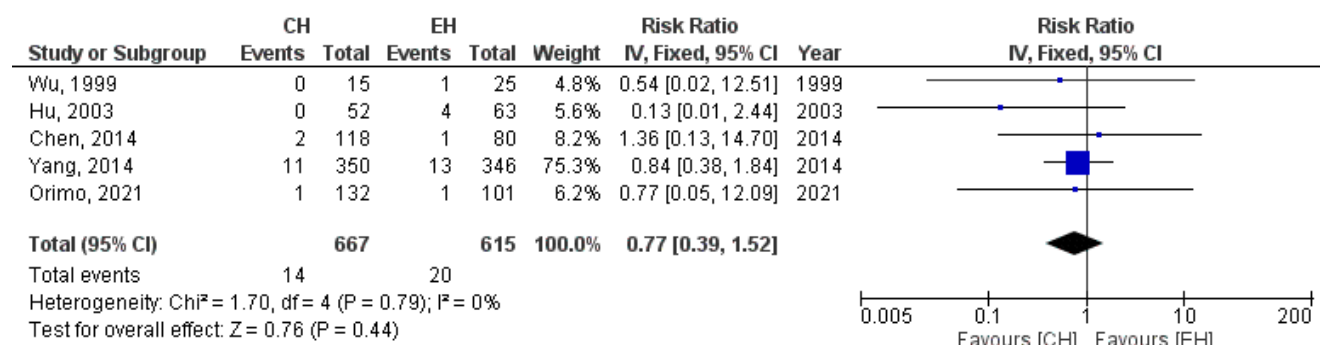

supplementary figure 6: Wound infection for CH and EH

Supplement: Supplementary file 6 — Additional file 6: Fig. S6. Wound infection for CH and EH. [file 12893_2022_1891_MOESM6_ESM.pdf]

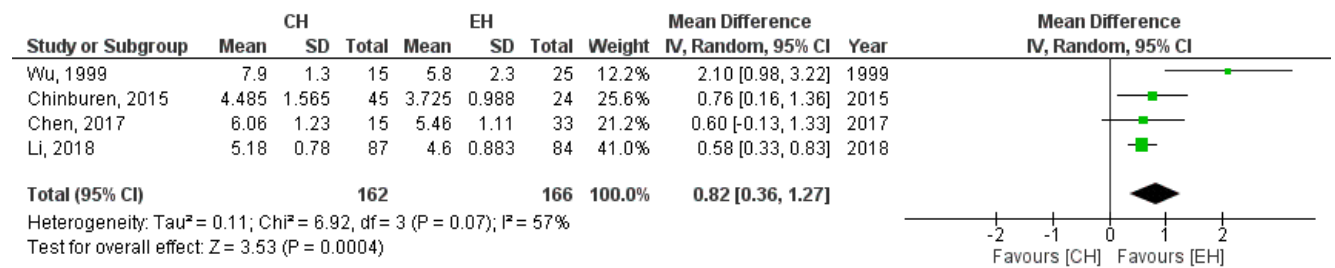

supplementary figure 7: Operative time for CH and EH

Supplement: Supplementary file 7 — Additional file 7. Fig. S7. Operative time for CH and EH. [file 12893_2022_1891_MOESM7_ESM.pdf]

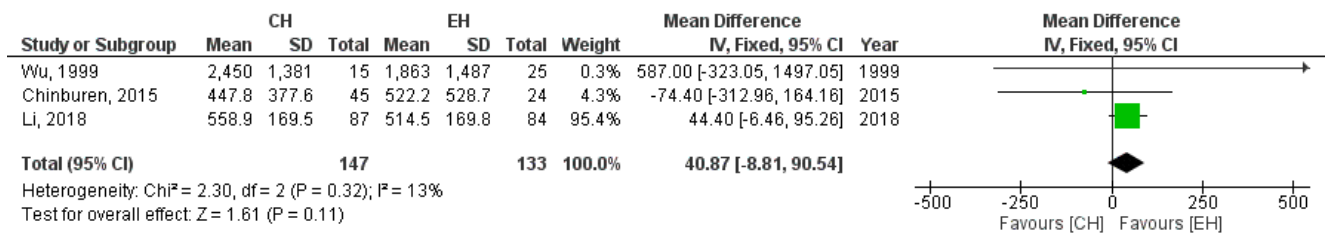

supplementary figure 8: Blood loss for CH and EH

Supplement: Supplementary file 8 — Additional file 8. Fig. S8. Blood loss for CH and EH. [file 12893_2022_1891_MOESM8_ESM.pdf]

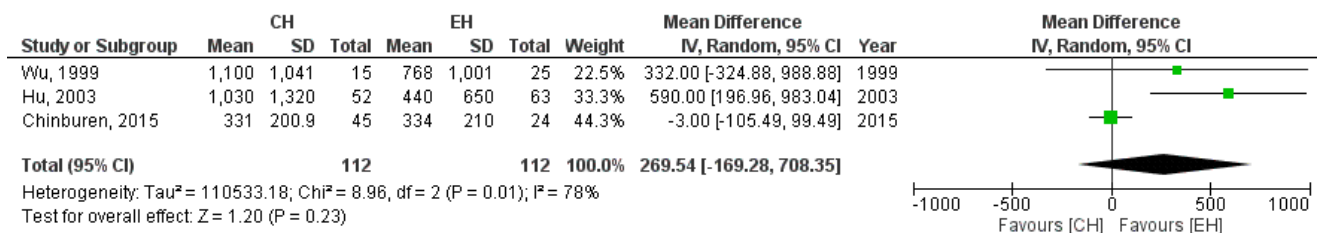

supplementary figure 9: Blood transfusion for CH and EH

Supplement: Supplementary file 9 — Additional file 9. Fig. S9. Blood transfusion for CH and EH. [file 12893_2022_1891_MOESM9_ESM.pdf]

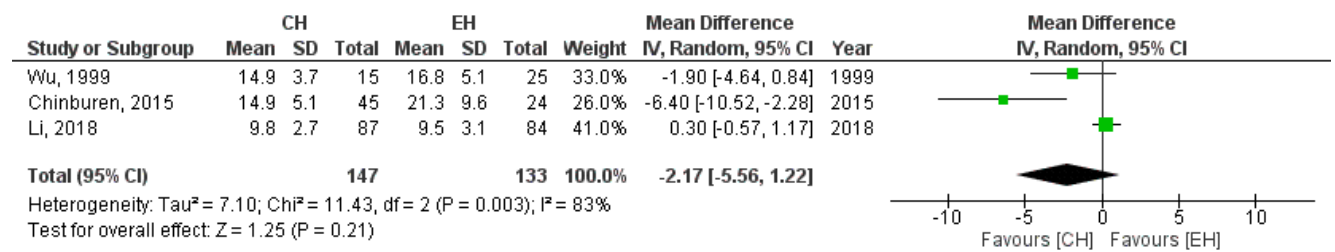

supplementary figure 10: Hospital stay for CH and EH

Supplement: Supplementary file 10 — Additional file 10: Fig. S10. Hospital stay for CH and EH. [file 12893_2022_1891_MOESM10_ESM.pdf]
